# Intramuscular Electrical Stimulation to Trigger Points: Insights into Mechanisms and Clinical Applications—A Scoping Review

**DOI:** 10.3390/jcm11206039

**Published:** 2022-10-13

**Authors:** Thomas Perreault, Andrew Ball, Jan Dommerholt, Robert Theiss, César Fernández-de-las-Peñas, Raymond Butts

**Affiliations:** 1 Department of Physical Therapy, Wentworth Douglass Hospital, Dover, NH 03820, USA; 2 Atrium Health, Carolinas Rehabilitation, Charlotte, NC 28216, USA; 3 Myopain Seminars, Bethesda, MD 20814, USA; 4 Department of Physical Therapy and Rehabilitation Science, School of Medicine, University of Maryland, Baltimore, MD 21201, USA; 5 Department of Physical Medicine and Rehabilitation, Atrium Health, Carolinas Rehabilitation, Charlotte, NC 28203, USA; 6 Department of Physical Therapy, Occupational Therapy, Rehabilitation and Physical Medicine, Universidad Rey Juan Carlos, 28922 Madrid, Spain; 7 Cátedra Institucional en Docencia, Clínica e Investigación en Fisioterapia: Terapia Manual, Punción Seca y Ejercicio Terapéutico, Universidad Rey Juan Carlos, 28922 Madrid, Spain; 8 Department of Rehabilitative Sciences, Gannon University, Ruskin, FL 33573, USA

**Keywords:** electrical stimulation, intramuscular, trigger point, myofascial pain

## Abstract

Intramuscular electrical stimulation (IMES) is a modality used by clinicians to treat myofascial pain. Recent studies have shown positive results for the use of IMES on pain outcomes, yet studies investigating the potential mechanisms of IMES directly to trigger points (TrPs) are lacking. We performed a scoping review of the literature to summarize the current evidence from human and animal studies on the mechanisms of IMES to the TrP location, and to identify gaps in the existing literature. Electronic literature searches were conducted across five databases from inception to 15 August 2022, including PubMed, Cumulative Index to Nursing and Allied Health Literature (CINAHL), Allied and Complementary Medicine Database (AMED), Scopus and Cochrane Register of Controlled Trials. Four studies met our full criteria for inclusion in this review. Three studies assessed the effects of IMES to TrPs on human subjects with MPS, and only one study used an animal model with experimentally generated TrPs. Based on the results of the included studies, IMES within a TrP region was reported to normalize muscle blood flow, decrease endplate noise of the TrP and elicit antinociceptive effects, at least partially, through engaging supraspinal descending pain inhibitory systems. At present, no clinical implications can be determined on the use of IMES to TrPs due to the limited amount and quality of the available evidence. Further studies investigating the clinical effectiveness and also underlying mechanisms of IMES to TrPs are clearly needed.

## 1. Introduction

Myofascial pain syndrome (MPS) is a prevalent condition routinely treated by healthcare practitioners [1]. It is characterized by the presence of trigger points (TrPs), which are painful spots within taut bands of muscle that produce local and referred pain either spontaneously or upon stimulation [2]. Trigger points are prevalent across a broad range of musculoskeletal conditions [3,4,5,6,7,8,9,10] along with certain neurological disorders [11,12,13] as well as breast cancer [14,15]. Studies suggest that alterations in blood flow arise in TrPs due to compression of blood vessels near or within the taut band [16,17], leading to focal regions of hypoperfusion and hypoxia within a TrP [18]. A recent study found foci of segmentally contracted sarcomeres, i.e., contracture knots, in the vicinity of a taut band and TrP region [19]. These focal areas of sustained contraction are suggested to be hypo-perfused in relation to the surrounding area [20]. Because of reduced blood flow, Shah et al. suggest that TrP pain is induced from elevated levels of bioactive substances and acidity accumulating in the TrP region [21,22]. Therapeutic interventions that enhance blood flow to the TrP are recommended [23,24].

Evidence for conservative management of MPS has increased and is trending toward a multimodal approach [25,26,27,28]. Common interventions include manual TrP therapy [29], exercise [30,31], transcutaneous electrical nerve stimulation (TENS) [32], and needling interventions, such as dry needling [33] and acupuncture [34]. Pharmacological treatments include nonsteroidal anti-inflammatory drugs (NSAIDs), try-cyclic antidepressants, muscle relaxants, local anesthetics, and botulinum toxin delivered via injection [35]. In addition, electrotherapeutic modalities using needles, such as percutaneous electrical nerve stimulation (PENS) and electroacupuncture (EA), are also reported to be effective for the management of myofascial pain [36,37,38,39]. These interventions share similarities with other forms of neuromodulation, i.e., they use electrical stimulation to modulate activity of neural pathways peripherally and centrally to decrease pain [40,41].

Intramuscular electrical stimulation (IMES) is an electrotherapeutic modality that uses needles to deliver electrical current into muscles and, more specifically, into TrPs [42]. The electrical stimulation is often used as an additive to manual needling. In practice, manual needle manipulation may be minimized in some situations and the needles may purely be used as electrodes to induce analgesic effects. Nevertheless, both electrical and manual stimulation of needles have overlapping physiological mechanisms due to the insertion of needles [43]. Evidence is gradually accumulating to support the use of IMES in patients with MPS [39,42,44,45,46]. However, a recent systemic review on IMES reported that among the six included studies, only three directly targeted TrPs [47]. Furthermore, objective variables related to the effects of IMES to the TrP region were lacking in all studies, limiting insight into the potential mechanisms of IMES. Ahmed et al. reported similar conclusions, suggesting that future studies should investigate the effects of electrical stimulation to specific anatomical regions in patients with MPS to better understand mechanisms of action [32].

Systematic reviews usually address clinical effectiveness of a therapeutic intervention to better guide clinical practice decisions based on randomized clinical trials. As it has been previously presented, there is a small number of clinical trials using IMES targeting TrPs [47]. In such a scenario, scoping reviews are used to identify, report, and discuss the available evidence on a specific topic/concept; an appraisal procedure usually referred to as “evidence mapping”. In fact, scoping reviews can be used for discussing current evidence on mechanisms and other topics which are not commonly assessed in systematic reviews or meta-analyses. Accordingly, scoping reviews are ideal tools to map current evidence of emerging topics by providing an indication for future research in those circumstances where systematic reviews or meta-analyses are unable to meet the necessary objectives of knowledge users. To the best of the authors knowledge, no review has been published yet investigating the mechanisms of IMES to the TrP location with inclusion of both human and animal studies. Therefore, the aim of our scoping review is to summarize current evidence from studies on the mechanisms of IMES to the TrP location.

## 2. Methods

### 2.1. Scoping Review

This scoping review followed the methodological framework for scoping reviews outlined by the Preferred Reporting Items for Systematic Reviews and Meta Analyses Extension for Scoping Reviews (PRISMA-ScR) [48]. This scoping review is registered on Open Science Framework (https://osf.io/rkdc3 accessed on 11 August 2022).

### 2.2. Search Strategy

Electronic literature searches were conducted across five databases from inception to 15 August 2022: PubMed, Cumulative Index to Nursing and Allied Health Literature (CINAHL), Allied and Complementary Medicine Database (AMED), Scopus, and Cochrane Register of Controlled Trials. Literature searches were conducted between 12 August 2022 and 15 August 2022. The search syntax was formulated with the collaboration of an experienced health science librarian. No restrictions were placed on publication date or language. In the search formulas, the following terms were merged using the following Boolean operators: “electrical stimulation”, electroacupuncture, “electro acupuncture”, electric*, intramuscular, percutaneous, “dry needling”, acupuncture, needling, myofascial, “trigger point”, “trigger spot”, muscular, muscle, and nerve. Additionally, we manually screened the reference lists of included papers, performed backward citation searching and used the “similar articles” feature on PubMed to identify additional studies. All database search strategies are outlined in Table 1. 

### 2.3. Study Selection

Two authors independently reviewed titles and abstracts of all identified articles for potential eligibility for full-text review. Two investigators were required to achieve a consensus on studies included for full text review or study inclusion. In the event of conflict between both reviewers, a third author independently participated to reach consensus for inclusion or exclusion for the study. Studies were included or excluded based on the criteria displayed in Table 2.

### 2.4. Data Mapping

Covidence systematic review software (Veritas Health Innovation, 2021) is an online tool we used to organize the current scoping review. Following the search period, all citations identified from each database search were imported to Covidence for automatic removal of duplicates, to begin initial screening of titles and abstracts, to determine studies eligible for inclusion following full text review, and inclusion or exclusion based on our criteria. The following data from each of the included studies was extracted using tables in Covidence: authors, year of publication, study population, condition, TrP diagnostic criteria, IMES parameters, outcomes, and mechanisms assessed. After extracting and interpreting the data from each study, we organized the topics thematically as effects of interventions, trigger point diagnosis criteria, intramuscular electrical stimulation paraments, and mechanisms of intramuscular stimulation.

## 3. Results

### 3.1. Search Results

**Figure 1:**
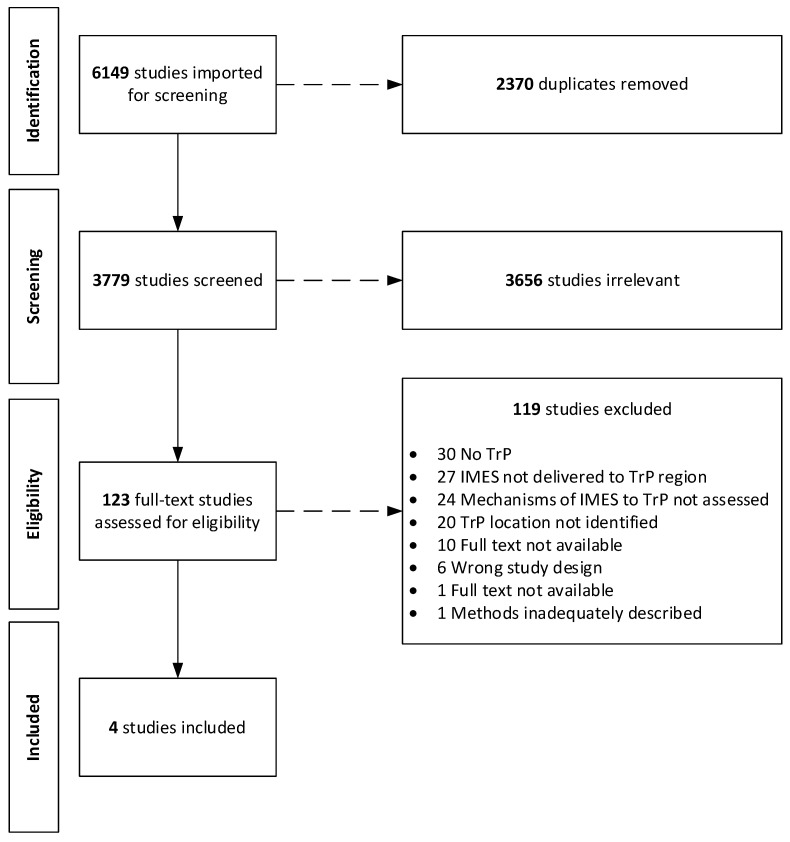
Flow diagram of search process based on Preferred Reporting Items for Systematic Reviews and Meta Analyses Extension for Scoping Reviews (PRISMA-ScR).

We retrieved 6149 studies, and after removal of duplicates, 3779 remained. Following the screening of titles and abstracts, a total of 123 studies were chosen for full-text review. One hundred and nineteen (*n* = 119) studies were excluded for reasons stated in Figure 1. Finally, four studies met our full criteria for inclusion in this review [49,50,51,52].

### 3.2. Study Population

Three of the included studies assessed the effects of IMES to TrPs on individuals with MPS [49,50,51]. Upper trapezius TrPs were treated in two studies [50,51], one study treated TrPs in either the levator scapulae or upper trapezius [49]. Only one study used an animal model and investigated IMES to the levator auris longus muscle in a TrP that was generated by subcutaneous injection of neostigmine [52].

### 3.3. Effects of Interventions

While the intent of this scoping review was to investigate mechanistic effects of IMES to the TrP, the results on pain outcomes are mentioned. Table 3 summarizes the effects of IMES treatment on pain outcomes. Lee et al. demonstrated a significant improvement in pain on the VAS (0–10 cm scale) and increases in pressure pain threshold (PPT) over the TrPs immediately after IMES and at the end of the study [49]. This study used an open-label, before-and-after treatment design with individuals receiving 4 sessions of IMES over 4 weeks. Muller et al. reported a significant reduction in general and localized upper trapezius pain on VAS (0–10 cm) in the EA treatment group and in the comparison group who received only manual acupuncture. Both groups received 8 treatment sessions of 30 min duration [50]. Niddam et al. reported that 10 out of 21 patients had significantly increased pain threshold to IMES in TrPs of the upper trapezius following intervention. More specifically, the intensity of IMES in mA applied to TrPs required to elicit patient-reported pain was elevated following treatments in some patients. Likewise, PPTs over the TrPs were shown to increase significantly in 12 out of 21 patients [51]. Margalef et al. did not assess clinical outcomes related to pain and used an animal model [52].

### 3.4. Trigger Point Diagnostic Criteria

Only two of the four studies reported the use of specific criteria for TrP diagnosis [50,51]. Mueller et al. used the most robust criteria for TrP diagnosis [50] to include a palpable tender spot within a taut band of muscle, local twitch response elicited by the snapping palpation of the taut band, reproduction of a referred pain pattern, and patient familiar pain reproduced upon TrP compression. Trigger points were considered active if the clinical complaint was reproduced with TrP stimulation; if not, they were considered latent. Niddam et al. used elicitation of a local twitch response by manipulation of a needle electrode in the TrP as one criterion for TrP diagnosis. In addition, the presence of a palpable band or hardened nodules within the upper left trapezius muscle and pain emanating from a well localized area (TrP) in the palpable band were used [51]. Lee et al. did not report any specific criteria for TrP diagnosis. Subjects were included if they had active TrPs in the unilateral upper trapezius or levator scapulae region, and had reliable symptoms caused by TrP palpation [49]. Following injection of neostigmine, Margalef at al. used palpation in order to identify a taut band within an area of muscle, needle EMG to identify end plate noise, and production of a local twitch response (LTR) under US imaging to locate TrPs [52].

### 3.5. Intramuscular Electrical Stimulation Parameters

All of the included studies used low-frequency electrical stimulation to TrPs within a range of 2–10 Hz (Table 4). However, Mueller et al. alternated low (2 Hz) and high (100 Hz) frequency for 5 s each in their protocol [50]. The intensity of stimulation varied greatly between studies ranging between 0.4 mA to upwards of 20 mA. Muller et al. did not report exact values for the stimulation intensity, instead they reported using maximal painless stimuli until a muscle contraction was observed [50]. Niddam et al. applied “mildly painful” stimulation intensity throughout their experiment with intensity ranging between 3.3–6.1 mA [51]. Pulse width was set at 1 ms in two studies [49,51], one study used continuous direct current for 3–5 s durations [52], and one study used pulse widths of 500–700 µs [50]. The treatment duration also varied greatly between studies, with two studies applying IMES for 3 min [49,51], one study for 10 min [52], and one study for 30-min durations [50]. The number of sessions ranged between 2–8.

### 3.6. Mechanisms of Intramuscular Electrical Stimulation

In those studies assessing mechanisms of IMES delivered to TrPs, two studies reported increased blood flow following the intervention [49,50] and one study reported effects on the descending pain inhibitory system [51]. In the single study using an animal model, the effects of IMES on endplate noise (EPN) at the TrP was assessed [52]. Lee et al. reported significant increases in regional muscle blood flow following low-frequency IMES applied to upper trapezius TrPs using laser doppler flowmetry through a surface probe over the TrP area [49]. Müller et al. reported improvements in local blood flow following EA using alternating 2 Hz and 100 Hz frequencies over eight sessions of 30-min duration. In this study, observations were performed under two-dimensional ultrasound (US) and ultrasound elastography, demonstrating reductions in the size of TrPs and reductions in muscle stiffness, respectively, even though changes in the latter were non-significant [50]. Using fMRI, Niddam et al. found enhanced activation in the dorsal periaqueductal gray (PAG) following 2 Hz IMES for 3-min durations within TrPs of the upper trapezius. Importantly, increased PAG activity was correlated with increases in PPT following IMES intervention in responders (i.e., patients achieving increases of twice the standard deviation of individual preintervention PPT) [51]. Using electromyography (EMG), Margalef et al. reported that percutaneous electrical current to the TrP area antagonized the effects of neostigmine injection, normalizing the EPN in the TrP areas. In addition, compared to dry needling alone, the use of electrical currents increased the total number and speed of local twitch responses (LTRs), by 144% and 230%, respectively. Applications of a higher intensity current of 3 mA, compared to 1.5 mA or 0.4 mA, proved most effective [52].

## 4. Discussion

To our knowledge, our scoping review using the PRISMA-ScR methodological framework is the first to investigate the mechanisms of IMES to the TrP location, and therefore is an important contribution to the literature. According to the results of our scoping review, and previous reviews [32,47], few studies investigating the mechanisms of IMES to the TrP are available. Clinical studies suggest the addition of electrical current to dry needling or acupuncture is an effective treatment option for MPS, but few have also specifically targeted the TrP. We identified moderate variation between the included studies regarding parameters of electrical stimulation. Yet, every study employed use of low-frequency electrical stimulation ranging between 2–10 Hz, and most studies used a moderate stimulation intensity to the TrP. Most importantly, the mechanistic effects of IMES to the TrP region were found to revolve around mitigating the EPN associated with the TrP or taut band, normalizing blood flow in the treated muscle and activating descending pain inhibitory systems. Currently, clinical implications regarding the use of IMES to TrPs are unclear due to the limited quantity and quality of the available evidence.

In the following sections, we further discuss the potential mechanisms of IMES to the TrP with supporting evidence.

### 4.1. Electrophysiological Mechanisms

In the study by Margalef et al., percutaneous electrical stimulation to the TrP reduced the number of areas and frequency of the EPN, as measured by needle EMG. Using ultrasound imaging, the speed and number of LTRs were observed to increase during applications of electrical currents compared to manual needle manipulation alone [52]. According to the authors, the steady depolarization of axon terminals combined with the production of LTRs elicited by the electrical stimulation, may have depleted acetylcholine (ACh) levels via repeated local muscle contraction. Endplate noise, also defined under spontaneous electrical activity (SEA), at the TrP is due to abnormally increased amounts of spontaneously released ACh at the extrafusal motor endplate [53]. The excess ACh is theorized to increase motor endplate activity, contributing to the formation of the taut band via local muscle fiber contractions [54,55]. Kuan et al. reported a positive correlation between the prevalence of EPN and the subjective pain intensity of TrPs in the upper trapezius. They also found that the prevalence of EPN was inversely correlated with the PPT in patients with MPS [56]. Similarly, pain intensity and the prevalence of LTRs during needling of TrPs have been found to be highly correlated [57]. 

In a recent study, dry needling to trigger spots (TrSs) in rats was shown to decrease the EPN at the TrS site, with corresponding decreases in the concentrations of ACh, and increases in acetylcholinesterase [58]. A recent study on human subjects revealed that dry needling under needle EMG guidance significantly reduced pain along with the amplitude and frequency of EPN at the TrP, compared to a control group that had dry needling performed without needle EMG guidance [59]. Both studies suggest that precise needling within the TrP results in greater TrP inactivation due to a reduction in ACh levels, and therefore pain relief. Thus, it is conceivable that like dry needling, IMES to a TrP elicits some of its therapeutic effects by normalizing EPN at the motor endplate. Moreover, use of electrical current applied to the inserted needles instead of repeated needle punctures required of manual needle manipulation to stimulate a TrP may reduce local muscular injury, inflammatory reactions, and post-needling soreness [60,61,62,63]. 

### 4.2. Blood Flow Mechanisms

**Figure 2:**
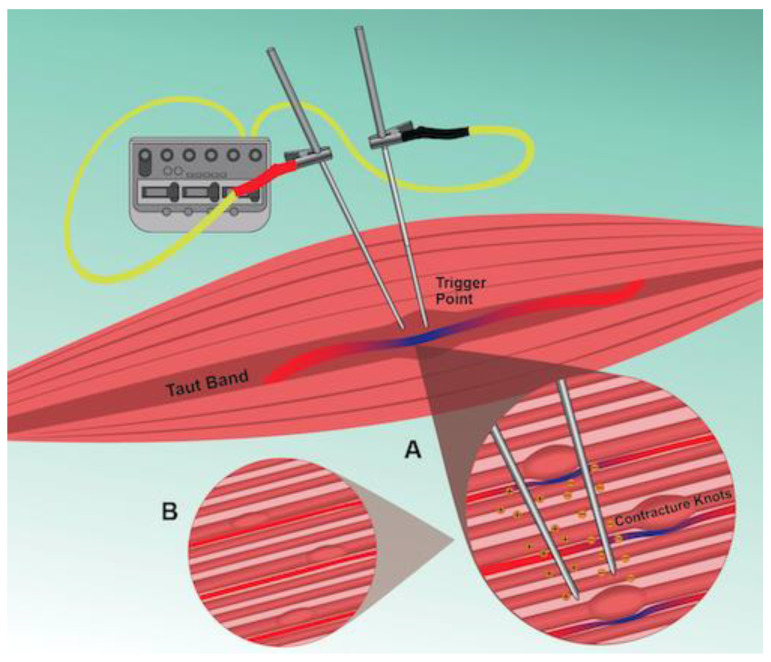
Schematic illustration of IMES to the TrP region. A: Delivery of a negative electrical charge into the TrP alters the potential difference across nearby neural membranes. Depending on the stimulation intensity, nerve depolarization, and production of compound action potentials will occur along motor and sensory nerve fibers. Local muscle contractions elicited by activation of motor fibers, or other reflex mechanisms, lead to vasodilation of blood vessels near or within the TrP. B: The increased microcirculation, and the prolonged depolarization of axon terminals combined with the production of LTRs elicited by IMES leads to depletion of acetylcholine (ACh) levels and reduction of EPN at the TrP.

We located two studies reporting enhanced blood flow following IMES to TrPs, with neither study measuring blood flow changes intramuscularly. One study reported use of laser doppler flowmetry through a surface probe over the TrP [49], and the other reported improved microvascularization based on reductions in TrP size observed under two-dimensional ultrasound [50]. Therefore, it is difficult to discern whether microcirculatory changes occur in or around the TrP region following IMES. Yet, several additional studies have shown that electric stimulation applied percutaneously increases muscle blood flow [64] and muscle blood volume [65,66] and enhances muscle tissue oxygenation for short durations post treatment [67]. An earlier study demonstrated that the muscle contractions induced by EA are necessary to elicit vasodilation of blood vessels by stimulating the release of nitric oxide, leading to increased microcirculation after the intervention [68]. Another study reported that release of calcitonin gene-related peptide (CGRP) promotes increased blood flow to the targeted muscle following electrical stimulation proximally to the ipsilateral dorsal roots [69]. In addition, CGRP has been shown to promote increases in muscle blood flow locally following manual acupuncture [70]. Furthermore, EA to skeletal muscles in rats was shown to increase the expression of hypoxia-inducible factor-1α (HIF-1α), which may promote angiogenesis and assist with vasodilation under hypoxic conditions [71]. Likewise, dry needling to TrPs increases HIF-1α concentrations in the locally treated muscle in a dose-dependent manner [72]. Although the above-mentioned studies did not deliver IMES to the TrP, it is tempting to suggest that the observed effects would occur in the TrP area if the TrP was directly targeted. Collectively, studies on human and animal models support that IMES normalizes muscle blood flow, potentially offsetting local ischemic and hypoxia that contribute to the TrP pathophysiology (Figure 2).

### 4.3. Supraspinal Mechanisms

Only one included study suggested that IMES to TrPs elicited antinociceptive effects through engaging descending pain inhibitory systems. Niddam et al. [51] found enhanced activation in the dorsal PAG following IMES within TrPs. A recent study by Botelho et al. suggested that IMES has restorative effects on impaired pain modulation in patients with chronic MPS [73]. While TrPs were not directly targeted, enhanced serum levels of brain neurotrophic factor (BDNF) and attenuation of cortical hyperexcitability was demonstrated following IMES intervention [73]. Interestingly, brain imaging studies on patients with chronic MPS reveal atrophic changes in brain regions associated with descending pain modulation [74,75]. Niddam et al. found gray matter atrophy in the anterior hippocampus, the ventrolateral and dorsolateral prefrontal cortices, superior frontal gyri, claustra, and the middle and anterior temporal gyri [74]. The amount of gray matter atrophy correlated with significant reductions in PPTs over TrPs in the upper trapezius muscles. Using diffusion kurtosis imaging, Xie et al. identified microstructural damage within several brain regions associated with pain perception in patients with chronic neck pain related to TrPs in the trapezius muscles [75]. A recent systematic review supported that conservative therapies can increase gray matter volume in the dorsolateral prefrontal cortex and enhance its connectivity to the PAG, following an intervention period in patients with chronic musculoskeletal pain conditions [76]. It is conceivable that neuroplastic changes in pain pathways may be induced via IMES to TrPs leading to improved function of a potentially impaired descending pain modulatory system [77]. 

### 4.4. Strengths and Limitations

The strengths of this scoping review include a comprehensive literature search, strict inclusion and exclusion criteria, careful data extraction using Covidence software, and thorough reporting on results of identified studies through tables, figures, and discussion. However, our review has some limitations. First, the human studies identified that measuring blood flow changes did not assess the effects intramuscularly, and it is difficult to conclude if microcirculatory changes occur in or around the TrP region following IMES. In the Muller et al. study, blood flow changes were proposed based on US interpretation; however, as stated above, they were not internally measured [50]. Second, we also recognize that the study by Margalef et al. measured the effects of IMES on trigger points in mice that were created chemically through injection of neostigmine [52], which creates spontaneous neurotransmission that can be recorded by EMG. This scenario differs from what is seen in clinical practice, where patients harbor TrPs for longer periods and they develop TrPs by other means. Third, in addition to identifying that low-frequency IMES was used in each study, we could not make any other assumptions about specific parameters of IMES on the effects at the TrP. This was due to lack of comparison groups in the included studies that used alternate stimulation parameters. Further studies are needed that assess the effects of different IMES parameters on the TrP region. Fourth, only two of the four included studies compared mechanistic effects between electrical stimulation to needles and manual needling alone [50,52]. It should be considered that some of the mechanistic effects elicited by IMES to the TrP are partly due to the mechanical stimulation from the inserted needle. In our discussion, we described several studies supporting the mechanistic effects of manual needle stimulation within the relevant subsections, where possible. Future studies investigating mechanisms of IMES to the TrP should investigate the effects of both manual and electrical needle stimulation. Fifth, all of the included studies were of small sample size, and only one included study was a randomized controlled study. The only randomized controlled study by Müller et al. [50] was found to have good methodological quality based on the Physiotherapy Evidence Database (Pedro) Scale [78].

## 5. Conclusions

This scoping review located four studies that investigated mechanisms of IMES to TrPs. Studies on human subjects with MPS suggest that IMES to the TrP increases blood flow within the treated muscle and enhances activation in the dorsal PAG. In the single animal study included, IMES reduced EPN and demonstrated a greater ability to produce LTRs in the TrP compared to manual needling. Collectively, studies suggest that IMES increases muscle blood flow, potentially offsetting local ischemic and hypoxia that contribute to the TrP pathophysiology. In addition, IMES within the TrP may elicit some of its therapeutic effects by normalizing EPN at the motor endplate and improving function of the descending pain modulatory system. Currently, clinical implications regarding the use of IMES to TrPs are unclear due to the limited quantity and quality of the available evidence. Further research is needed to confirm the mechanistic effects of IMES to TrPs. 

## Figures and Tables

**Table 1:** Database formulas during literature search.

Database	Search Strategy	Studies Located
PubMed/ Medline	(“Electrical Stimulation”[Title/Abstract] OR electroacupuncture[Title/Abstract] OR “electroacupuncture”[Title/Abstract] OR electric*[Title/Abstract]) AND (Intramuscular[Title/Abstract] OR percutaneous [Title/Abstract] OR “dry needling”[Title/Abstract] OR Acupuncture[Title/Abstract] OR needling[Title/Abstract]) AND (myofascial[Title/Abstract] OR “trigger point”[Title/Abstract] OR “Trigger spot”[Title/Abstract] OR “contracture knot”[Title/Abstract] OR contraction knot[Title/Abstract] OR Muscular[Title/Abstract] OR muscle[Title/Abstract] OR nerve[Title/Abstract])	2323
CINAHL	TI ((“Electrical Stimulation” OR electroacupuncture OR “electro acupuncture” OR electric*)) OR AB ((“Electrical Stimulation” OR electroacupuncture OR “electro acupuncture” OR electric*)) AND TI ((Intramuscular OR percutaneous OR “dry needling” OR Acupuncture OR needling)) OR AB ((Intramuscular OR percutaneous OR “dry needling” OR Acupuncture OR needling)) AND TI ((myofascial OR “trigger point” OR “Trigger spot” OR Muscular OR muscle OR nerve)) OR AB ((myofascial OR “trigger point” OR “Trigger spot” OR Muscular OR muscle OR nerve))	736
AMED	TI ((“Electrical Stimulation” OR electroacupuncture OR “electro acupuncture” OR electric*)) OR AB ((“Electrical Stimulation” OR electroacupuncture OR “electro acupuncture” OR electric*)) AND TI ((Intramuscular OR percutaneous OR “dry needling” OR Acupuncture OR needling)) OR AB ((Intramuscular OR percutaneous OR “dry needling” OR Acupuncture OR needling)) AND TI ((myofascial OR “trigger point” OR “Trigger spot” OR Muscular OR muscle OR nerve)) OR AB ((myofascial OR “trigger point” OR “Trigger spot” OR Muscular OR muscle OR nerve))	298
Scopus	((TITLE ((“Electrical stimulation” OR electroacupuncture OR “electro acupuncture” OR electric*)) OR ABS ((“Electrical Stimulation” OR electroacupuncture OR “electroacupuncture” OR electric*)))) AND ((TITLE ((intramuscular OR percutaneous OR “dry needling” OR acupuncture OR needling)) OR ABS ((intramuscular OR percutaneous OR “dryneedling” OR acupuncture OR needling)))) AND ((TITLE ((myofascial OR “trigger point” OR “Trigger spot” OR muscular OR muscle OR nerve)) OR ABS ((myofascial OR “trigger point” OR “Trigger spot” OR muscular OR muscle OR nerve)))) AND (LIMIT-TO (DOCTYPE, “ar”) OR LIMIT-TO (DOCTYPE, “re”) OR LIMIT-TO (DOCTYPE, “cp”))	1884
Cochrane Central Register of Controlled Trials	TI ((“Electrical Stimulation” OR electroacupuncture OR “electro acupuncture” OR electric*)) OR AB ((“Electrical Stimulation” OR electroacupuncture OR “electro acupuncture” OR electric*)) AND TI ((Intramuscular OR percutaneous OR “dry needling” OR Acupuncture OR needling)) OR AB ((Intramuscular OR percutaneous OR “dry needling” OR Acupuncture OR needling)) AND TI ((myofascial OR “trigger point” OR “Trigger spot” OR Muscular OR muscle OR nerve)) OR AB ((myofascial OR “trigger point” OR “Trigger spot” OR Muscular OR muscle OR nerve))	908

**Table 2:** Inclusion and exclusion criteria.

	Inclusion Criteria	Exclusion Criteria
Study design	Experimental or quasi-experimental studies randomized controlled trials, cohort studies, case series, case studies and animal studies	Systematic reviews, meta-analyses, scoping or narrative reviews, poster presentations
Interventions	Needle based electrotherapeutic modalities administered to the TrP region; Intramuscular Electrical Stimulation (IMES), Percutaneous Electrical Nerve Stimulation (PENS), Electroacupuncture (EA) or Electrical Dry Needling (DN)	Studies that did not administer electrical needle stimulation to an identified Trigger Point (TrP). Studies using Transcutaneous Electrical Nerve Stimulation (TENS), Neuromuscular Electrical Stimulation or only surface level electrical stimulation
Study population	Human or animal subjects with one or more TrPs diagnosed using consensus based-criteria and/or ultrasound, Electromyography (EMG), Magnetic Resonance Imaging (MRI), Magnetic Resonance Elastography (MRE) or other validated imaging to identify TrPs	Studies that did not report identifying the TrP location or that did not accurately locate the TrP according to inclusion criteria
Mechanisms evaluated	Studies assessing structural, biochemical, neurophysiological or brain change/function effects during or following needle based electrotherapeutic modalities administered to the TrP region. Mechanisms outcomes included, but were not limited to, microdialysis, electromyography, ultrasound, MRI/MRE, Functional Magnetic Resonance Imaging (fMRI), tissue biopsy, neuroanatomical tracing and intracellular recording.	Mechanisms not assessed or assessed but electrotherapeutic modalities administered to the TrP region
Language	English	Not English
Year of publication	No limits	No limits

**Table 3:** Description of clinical findings from included studies.

Study	Subjects	TrP Diagnosis	Clinical Findings
Lee et al. (2008) [49]	35 healthy females and 5 healthy males aged 33–51 with Myofascial Pain Syndrome (MPS)	Investigator identified most tender TrP and marked area.	Visual analog scale (VAS) and pressure pain thresholds (PPT) significantly improved immediately after each treatment. Immediate and mid-term positive effects on cervical and shoulder range of motion (ROM). Overall negative correlation between epidermal blood flow and VAS score before the first treatment.
Margalef et al. (2020) [52]	35 deceased young adult (45–50 days postnatal) male Swiss mice and 6 deceased young adult (60–70 days postnatal) Sprague Dawley rats	Levator auris longus was injected with neostigmine to promote ACh release and mimic TrPs in this region.	Applications of a higher intensity current (3 mA and 1.5 mA) proved most effective in reversing the action of neostigmine at both 3 h and 24 h. Application of percutaneous currents produced both an increase in the number and speed of local twitch responses compared with dry needling.
Muller et al. (2015) [50]	24 women with BMI 18–27.5 and regular menstrual cycle aged 20–40 with MPS	Blinded, experienced PT conducted palpation protocol and used 5 diagnostic criteria. Categorized into active or latent TrPs.	Significant decrease of intensity in general, right, and left trapezius pain VAS (0–10 cm) in the electroacupuncture group.
Niddam et al. (2007) [51]	13 healthy females and 8 healthy males with an average age of 35 and MPS	Palpable, painful band was identified and local twitches were evoked by needle manipulation.	48% of patients had a significant increase in pain threshold after IMES. 57% of patients had a significant increase in PPT.

**Table 4:** Summary of individual IMES parameters and mechanisms assessed.

Study	Muscles Treated	Frequency (Hz) Intensity (mA) Pulse Width	Number of Needles Sessions Duration (Mins)	Mechanisms Studied
Lee et al. (2008) [49]	Upper Trapezius or Levator Scapulae	2 Hz 0–20 mA based on visible contraction 1 ms	1 4 sessions 3 min	Regional blood flow significantly increased immediately but temporarily after each treatment.
Margalef et al. (2020) [52]	Levator auris longus and gastrocnemius	Protocol 1: 0.4 mA for 5 s Protocol 2: 1.5 mA for 5 s Protocol 3: 3 mA for 3 s Microcurrent stimulation: 10^−6^ mA at 10 Hz	3 per muscle 4 sessions in protocol 1, 3 sessions in protocol 2, and 3 sessions in protocol 3 10 min	Significant reduction of endplate noise in TrP areas following Intramuscular Electrical Stimulation (IMES). Total number and speed of LTRs increased by 144% and 230%, respectively, with IMES
Muller et al. (2015) [50]	Upper trapezius	Alternated between 2 Hz/700 µs/5 s and 100 Hz/500 µs/5 s	4 8 30 min	Decreased TrP area and strain ratio in both right and left trapezius post-treatment.
Niddam et al. (2007) [51]	Upper left trapezius	2 Hz 3.3–6.1 mA 1 ms	1 2 sessions in group 1, 3 sessions in group 2 3 min	IMES modulated PAG activity to painful stimuli more in patients that responded

## Data Availability

Not applicable.
